# Shape changes of erythrocytes during blood clot contraction and the structure of polyhedrocytes

**DOI:** 10.1038/s41598-018-35849-8

**Published:** 2018-12-17

**Authors:** Valerie Tutwiler, Alexander R. Mukhitov, Alina D. Peshkova, Giang Le Minh, R. R. Khismatullin, Jacqueline Vicksman, Chandrasekaran Nagaswami, Rustem I. Litvinov, John W. Weisel

**Affiliations:** 1 Department of Cell and Developmental Biology, University of Pennsylvania School of Medicine, Philadelphia, PA USA; 2 Institute of Fundamental Medicine and Biology, Kazan Federal University, Kazan, Russian Federation; 3 Department of General Pathology, Kazan State Medical University, Kazan, Russian Federation

## Abstract

Polyhedral erythrocytes, named polyhedrocytes, are formed in contracted blood clots and thrombi, as a result of compression by activated contractile platelets pulling on fibrin. This deformation was shown to be mechanical in nature and polyhedrocytes were characterized using light and electron microscopy. Through three-dimensional reconstruction, we quantified the geometry of biconcave, intermediate, and polyhedral erythrocytes within contracting blood clots. During compression, erythrocytes became less oblate and more prolate than the biconcave cells and largely corresponded to convex, irregular polyhedra with a total number of faces ranging from 10 to 16. Faces were polygons with 3 to 6 sides. The majority of the faces were quadrilaterals, though not all sides were straight and not all faces were flat. There were no changes in the surface area or volume. These results describe the gradual natural deformation of erythrocytes as a part of compaction into a tightly packed array that is an important but understudied component of mature blood clots and thrombi.

## Introduction

Erythrocytes were commonly believed to be passive bystanders in the processes involved in blood clotting, but recent studies revealed that they play a more influential role in both hemostasis and thrombosis^1,2^. Besides their indirect rheological effects, including marginalization of platelets in the blood flow, erythrocytes have the ability to directly modulate thrombin generation, fibrin formation, structure^3,4^, and the viscoelastic properties of the clot^3,5,6^. In particular, the incorporation of erythrocytes into the clot has been shown to determine the clot size^7^ and result in a hematocrit-dependent reduced extent of clot contraction (aka clot retraction)^5^. Clot contraction, or the volume shrinkage of the clot, occurs when activated platelets pull on the fibrin network^8,9^. This results in the compaction of erythrocytes to the core of the clots and redistribution of platelets and fibrin toward the outside of the clot^10^. The erythrocytes amassed in the core of the contracting clot undergo a shape transformation from their native biconcave shape to that of polyhedra, hence named polyhedrocytes^10^. The presence of polyhedrocytes in the core of the clot reduce clot permeability and consequently are thought to play a role in hemostasis^10^. The reduced extent of clot contraction, implying a larger volume and a less compact structure, may aggravate arterial and venous thrombosis due to increased obstructiveness and perhaps propensity of a thrombus to rupture and embolize^11,12^.

This remarkable polyhedral shape of erythrocytes, first described by Gottlob *et al*.^13^ and rediscovered later^10,12,14,15^, is a natural morphological form of erythrocytes in addition to echinocytes, acanthocytes, spheroechinocytes, ovalocytes, elliptocytes, stomatocytes, and more^16^. From a more general perspective, polyhedra have been observed in nature^17^ and in non-biological materials^18,19^ since before the time of Plato in a variety of plant and animal cells, viruses, tissues, foams and metals^20^. It has been shown that some polyhedral shapes allow for the minimization of interface area between cells^21^, the minimization of potential energy in the system^17^, and the stabilization of the membrane skeleton^22^. It is biologically important that polyhedrocytes are able to minimize the space between cells, due to more efficient packing, which helps to create an impermeable seal at the site of vessel injury to prevent bleeding^10,23^. Polyhedrocytes formed intravitally have been observed in clots and thrombi obtained from human and murine samples^10,12,23–25^.

Erythrocytes are able to undergo dramatic changes in shape due to their highly deformable nature^26^. Physiologically, erythrocytes must change from their native biconcave shape to a bullet-like shape every time they squeeze through the microcirculation to maintain a high surface area necessary for efficient exchange of oxygen and carbon dioxide^16^. Erythrocyte shape is determined by their viscosity, biconcave shape, and remarkably soft cytoskeleton^27^. The spectrin molecular network tethered to the phospholipid membrane of the erythrocytes provides the mechanical properties needed for the erythrocytes to easily deform under the application of force^28^. During the contraction process, the erythrocytes are exposed to compressive forces generated by the platelets and propagated by the fibrin network, which result in their compaction into the core of the clot and the formation of polyhedrocytes. Platelets have been shown to have an elastic modulus of 32 kPa in their center and 224 kPa at their periphery, due to the localization of the cytoskeleton at the edge of the cell^29^. Erythrocytes have been shown to have an elastic modulus ranging from several hundred Pa to a few kPa, making platelets much stiffer than erythrocytes^30^. Individual platelets are able to generate forces of ~80 nN/platelet^5,31^ and integral contractile stress in a whole blood clot can reach can reach over 3000 dyn/cm^2^ ^9^, which considerably exceeds the 75–150 dyn/cm^2^ that is required to deform erythrocytes into polyhedral-like cells^10^. Despite the fundamental biological significance and perhaps a great pathogenic importance of the compressive deformation of erythrocytes during contraction of blood clots and thrombi, the transition of red cells from biconcave to polyhedral shape remains enigmatic.

Here, we used histology, transmission electron microscopy, scanning electron microscopy and confocal microscopy of *in vitro* blood clots to visualize and quantify the formation of compressed erythrocytes during the clot contraction process. We were able to determine that purely mechanical forces that mimic those generated by contracting platelets can reproduce formation of a variety of polyhedron-like erythrocytes. These erythrocytes taking on a fully polyhedral shape have an average of 13 faces with between 3–6 sides on each face. Gaining a more detailed understanding of the formation of mechanically compressed polyhedral-like erythrocytes has the potential to shed light on the biomechanical and biological properties of contracted intravascular or extravascular clots and thrombi.

## Results

### Formation of polyhedral-like erythrocytes in contracted blood clots

**Figure 1:**
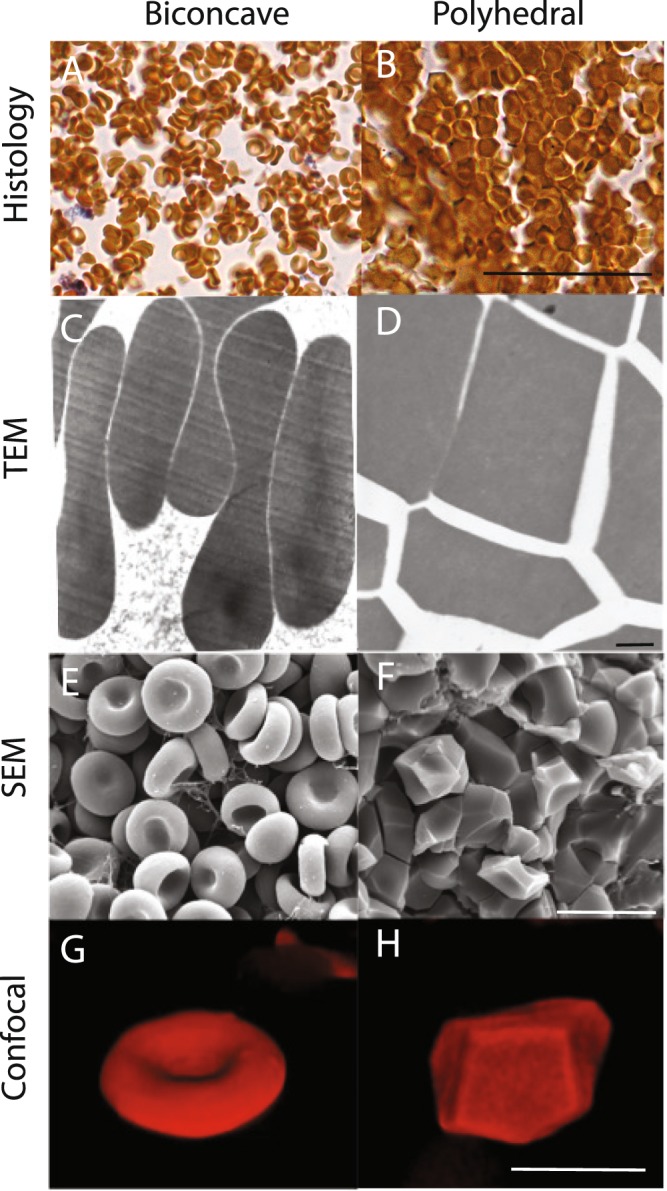
Visualization of biconcave erythrocytes and polyhedral erythrocytes (polyhedrocytes) in uncontracted and contracted blood clots, respectively. (**A**,**B**) Stained histological section (**C**,**D**) transmission electron microscopy (TEM), (**E**,**F**) scanning electron microscopy (SEM), and (**G**,**H**) confocal microscopy were used to image biconcave erythrocytes and polyhedrocytes. Scale bar is 25 µm. for histology, 1 µm for TEM, 10 µm for SEM, and 10 µm for confocal.

Because contraction of blood clots imposes mechanical load on erythrocytes^6^, we used four independent imaging modalities to assess the shape change of erythrocytes packed into the core of contracted clots. The shape transitions from biconcave to polyhedral were observed when assessing images from light microscopy (histological slides and confocal sectioning), transmission electron microscopy, and scanning electron microscopy (Fig. 1). Importantly, all of these methodologies reveal the formation of polyhedral-like cells following the clot contraction process, confirming that this shape change is a real biological process and not an artifact of sample preparation or imaging techniques. In *light microscopy* the stained clot sections contained two major types of erythrocytes. In the uncontracted clots (fixed within 2 minutes after addition of thrombin to whole citrated blood) only biconcave discoid erythrocytes were visualized (Fig. 1A), while in the contracted clots there were clearly visible tightly packed polygonal shapes (Fig. 1B). In *transmission electron microscopy* the cross-sections of individual erythrocytes were observed, where images of biconcave cells had a curved, generally elliptical cross-section (Fig. 1C) and polyhedrocytes (Fig. 1D) appeared as polygons that had defined edges on all sides of the cross-section. The *scanning electron microscopy* images allowed for one or more sides of the erythrocytes to be observed. The biconcave cells showed either a defined concave structure or a side view of the rounded circular part of the biconcave cell (Fig. 1E). Polyhedrocytes had clearly defined polygonal faces, with the type of polygon clearly distinguishable (Fig. 1F). Unlike the above imaging techniques, *confocal microscopy* allowed for the visualization of three-dimensional images of either biconcave cells (Fig. 1G) or polyhedrocytes (Fig. 1H), where all sides of the cell could be observed and quantified; the characterization of these images are described in more detail in following sections.

It is noteworthy that the erythrocytes observed were not all biconcave or polyhedral in nature, in that often we observed intermediate forms that had a mix of characteristics between the biconcave cells and the polyhedrocytes (Supplemental Fig. 1). These cells were characterized as either intermediate/biconcave or intermediate/polyhedral (Table 1, Supplemental Fig. 2). Clot contraction and the shape transformation of erythrocytes is a dynamic process, so histology images were used to determine that intermediate forms and polyhedrocytes begin to form between 5 and 15 mins following the onset of clotting (Supplemental Fig. 3). The influence of the clotting process on the presence of intermediate forms and polyhedrocytes is described in detail in the following sections.

**Table 1:** Morphological characteristics of erythrocytes within contracted blood clots from scanning electron micrographs.

Classification	Inclusion criteria
Biconcave	• round and smooth
	• no visible edges
	• pronounced dimple
Intermediate (mainly biconcave)	• round and smooth
	• no visible edges
	• no pronounced dimple/very faint dimple
Intermediate (mainly polyhedral)	• visible edges
	• wavy
	• no dimple
Polyhedral	• pronounced edges
	• flat faces
	• no dimple
	• tightly packed

**Figure 2:**
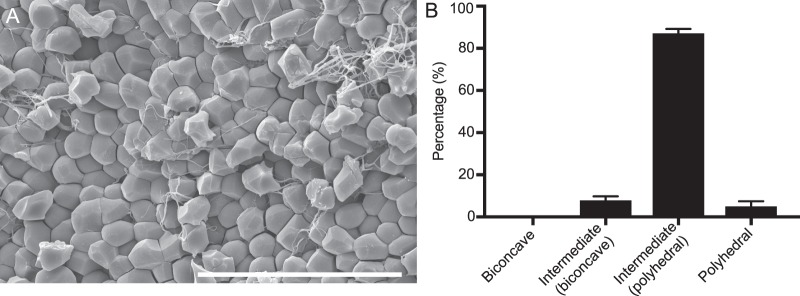
Erythrocyte shape change in contracted venous thrombi extracted from patients with deep vein thrombosis. (**A**) A representative scanning electron microscopy image of a venous thrombus showing formation of polyhedrocytes and intermediate shaped polyhedral-like RBCs. Scale bar is 30 µm. (**B**) Percentage of biconcave, intermediate and polyhedral erythrocytes in human venous thrombi (n = 9).

In addition to the observation of polyhedrocytes and intermediate forms in contracted blood clots, it is important to note that we determined that polyhedrocytes or intermediate/polyhedral cells make up 92 ± 2% of erythrocytes present in thrombi extracted from patients with deep vein thrombosis (Fig. 2). This finding points to the clinical importance of erythrocyte shape change not only in a physiological setting but also in understanding human pathologies.

### Erythrocytes’ polyhedron-like shape results from the mechanical forces

**Figure 3:**
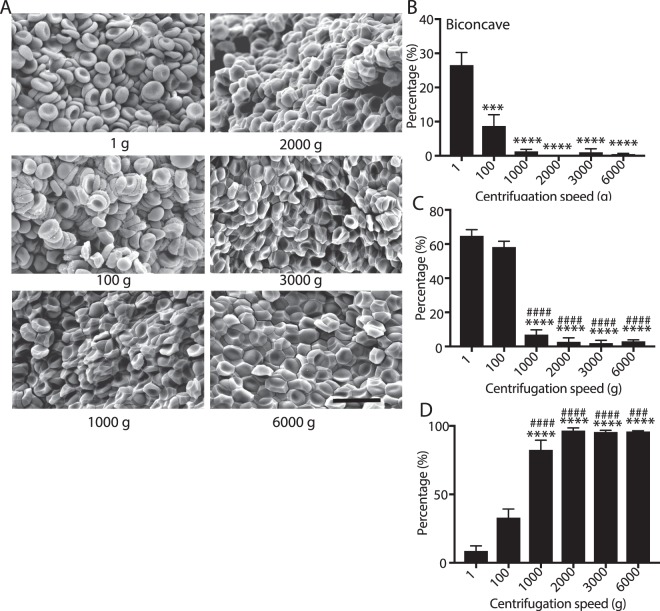
Effect of centrifugation on erythrocyte shape change. Washed erythrocytes were sedimented at 1 g to 6,000 g to examine the influence of mechanical deformation on shape changes. (**A**) Scanning electron microscopy was used to visualize the centrifuged erythrocytes. Scale bar is 10 µm. Scanning electron microscopy images were quantified for the percentage of (**B**) biconcave (**C**) intermediate (mainly biconcave), and (**D**) intermediate (mainly polyhedral). Statistical analysis was completed using a one-way ANOVA with an alpha value of 0.5 where significant differences are shown with respect to 1 g (*), 100 g. Data is represented as a mean ± SEM. N > 100 cells for each experimental condition.

**Figure 4:**
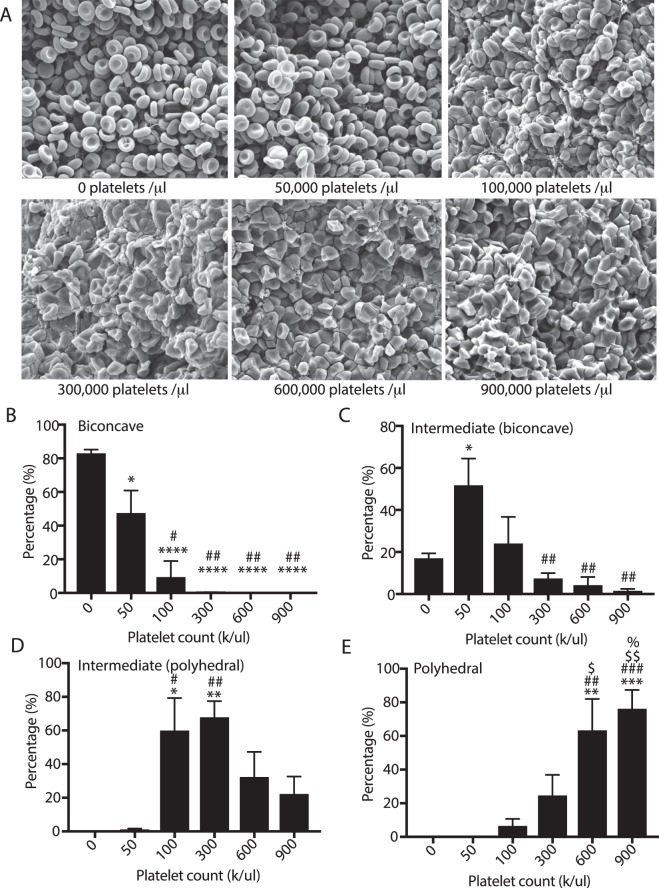
Influence of platelet count on formation of polyhedral-like erythrocytes in contracting blood clots. Reconstituted blood samples with platelet counts from 0 to 900,000/µl were activated with 1 U/ml thrombin and 2 mM CaCl_2_ allowed to clot and contract, and then imaged using scanning electron microscopy. (**A**) Representative scanning electron microscopy images showing that increased platelet count resulted in an increased fraction of polyhedral-like erythrocytes. Scale bar is 10 µm. Scanning electron microscopy images were quantified for the percentage of (**B**) biconcave, (**C**) intermediate (mainly biconcave), (**D**) intermediate (mainly polyhedral), and (**E**) polyhedral cells. Statistical analysis was completed using a one-way ANOVA with an alpha value of 0.5 where significant differences are shown with respect to no platelets (*), 50,000 platelets/µl (#), 100,000 platelets/µl ($), and 300,000 platelets/µl (%). Data is represented as a mean ± SEM. N > 100 cells for each experimental condition.

**Figure 5:**
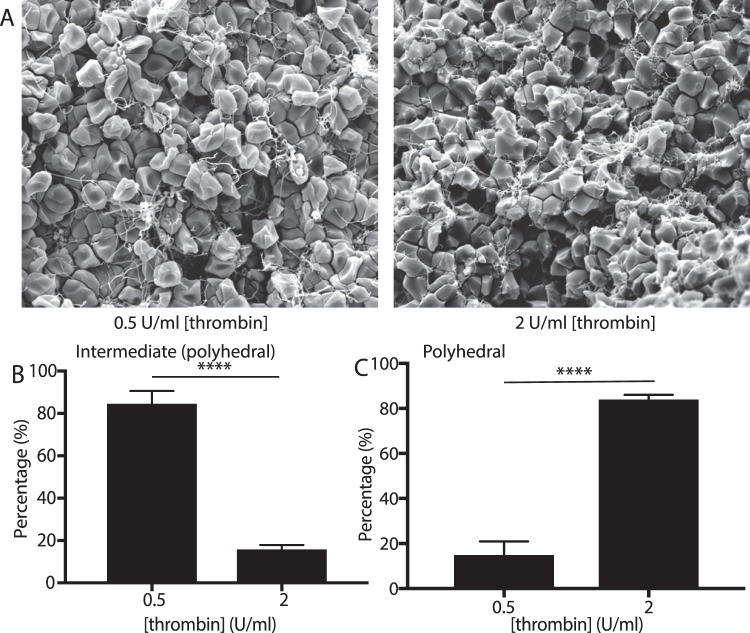
Influence of thrombin activity on formation of polyhedral-like erythrocytes in contracting blood clots. Reconstituted whole blood samples were activated with either 0.5 or 2 U/ml thrombin in the presence of 2 mM CaCl_2_ and allowed to contract and then imaged using scanning electron microscopy. (**A**) Representative scanning electron microscopy images showing that increased thrombin activity resulted in an increased fraction of polyhedral-like erythrocytes. Scale bar is 10 µm. Scanning electron microscopy images were quantified for the percentage of (**B**) intermediate (mainly polyhedral), and (**C**) polyhedral cells. Statistical analysis was completed using a one-way ANOVA with an alpha value of 0.5. Data is represented as a mean ± SEM. Between 400 and 600 erythrocytes were analyzed for each thrombin concentration.

Clot contraction is driven by a contractile, compressive pressure that pushes the erythrocytes toward the core of the clot^10,11^. To determine if production of polyhedrocytes is a purely mechanical process, we mimicked the contractile forces that are generated by platelets, which can generate forces over 3000 dyn/cm^2^ ^6,9^, using centrifugal forces applied to settled erythrocytes in the range of 1 g–6000 g. Erythrocyte shape change toward polyhedra was observed in citrated blood at accelerations as low as 100 g and tessellated, polyhedrocyte-like cells predominate at accelerations greater than 1000 g. The fraction of polyhedral-like cells increased with an increase of the applied centrifugal forces in the 100 g-1,000 g range (Fig. 3). Notably, there were few polyhedrocytes with flat sides that met the strict inclusion criteria (Table 1) likely because of the uniaxial application of the centrifugal forces resulting in a biomechanical mechanism of cell deformation distinct from uniformly applied compressive forces during clot contraction. From the weight (at an acceleration of 1 g) of a single erythrocyte of about 1 pN, the threshold stress needed to induce polyhedrocyte formation is estimated to be between 0.1 and 1 nN/m^2^ per erythrocyte, well below the range of stress generated during contraction, as individual platelets can generate forces at large as ~80 nN/m^2^ ^5,29^. Likewise, as activated platelets generate contractile force, there was a direct correlation between the extent of polyhedrocyte formation and increasing platelet count in the reconstituted blood samples with different platelet counts (Fig. 4) or with different concentrations of thrombin used as a platelet activator (Fig. 5). While we observed the marked erythrocyte shape changes at all volume fractions of erythrocytes, there was no correlation between the quantity of erythrocytes and the formation of polyhedrocytes (Supplemental Fig. 4). Taken together, these findings reveal that the erythrocyte shape change has a purely mechanical nature and depends on the amount of applied compressive pressure.

### Comparative volumetric characteristics of biconcave, intermediate, and polyhedral erythrocytes within a contracted clot

Because the volume and surface area of erythrocytes are paramount to their primary gas exchange function, we examined if the formation of polyhedrocytes resulted in changes in these characteristics. The volume and surface area were determined directly using three-dimensional reconstructions of the cells from contracted clots imaged with confocal microscopy. Only a small fraction (10%) of erythrocytes were stained, so that individual cells could be visualized and some cells near the edge of the clot were imaged, and others deeper into the core of the clot (~50 µm in depth; the clot dimensions were 6 mm wide by 3 mm high) were studied, which allowed for not only polyhedrocytes but also biconcave and intermediate forms of erythrocytes to be visualized and measured. Biconcave cells were typically observed closer to the edge of the clot while polyhedrocytes were observed toward the middle of the clot, and intermediate forms were observed throughout.

Nearly all cells, even the polyhedrocytes, had one dimension significantly smaller than the others, just as for the normal biconcave erythrocytes. This shorter dimension was called the height and measured for each cell. The dimensions in two directions perpendicular to the height were also measured.

The erythrocytes within a contracted clot were segregated into these three categories: biconcave, intermediate, and polyhedrocytes. In accordance with the inclusion criteria developed for the scanning electron microscopy images (Table 1, Supplemental Fig. 2), the biconcave cells were defined as being an oblate spheroid with two concave dimples. Polyhedrocytes were defined as cells where all faces of the cell were polygons. Intermediate forms were defined as having deviated from the biconcave shape and with some polyhedral features, but not all faces could be characterized as polygons, in that some faces were not flat, and some edges were not straight. ~70% of the erythrocytes analyzed by confocal microscopy were intermediate forms.

**Figure 6:**
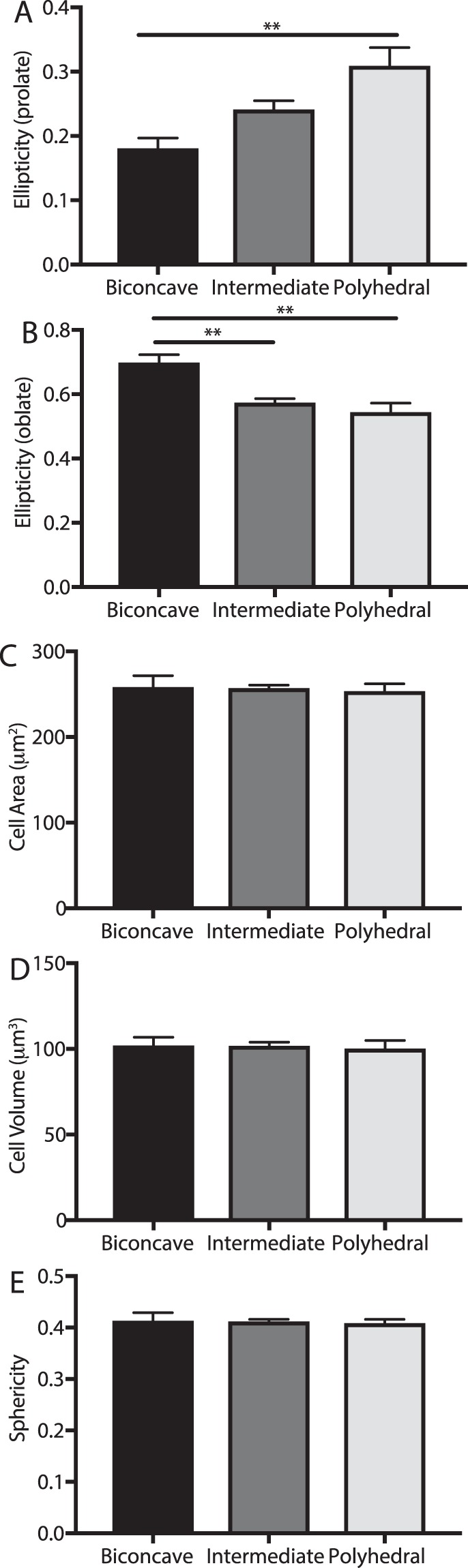
Analysis of volumetric properties of erythrocytes of various shapes. Three-dimensional reconstructions of biconcave (n = 12), intermediate (n = 86) and polyhedral (n = 22) erythrocytes from contracted whole blood clots were assessed for the (**A**,**B**) ellipticity, (**C**) cell area, (**D**) cell volume and (**E**) sphericity. One way ANOVA with an alpha of 0.05 was used to assess statistical significance, **corresponds to *P* < 0.01.

Intermediate forms and polyhedrocytes were both more prolate (Fig. 6D) than biconcave erythrocytes, and polyhedrocyes were less oblate than biconcave erythrocytes (Fig. 6E). Interestingly, the surface area and volume did not differ between cell categories (Supplemental Fig. 6A,B). This is consistent with the fact that there were no significant differences in the average height or other measured dimensions of the erythrocytes between cell categories (Supplemental Fig. 5A,B) The sphericity (Fig. 6C) and the height (Supplemental Fig. 5B) of the cells did not differ between cell categories, revealing that while the erythrocytes did become more elliptical, there were no changes in the surface area or volume of the cell.

### Comparative morphological characteristics of biconcave, intermediate, and polyhedral erythrocytes within a contracted clot

**Figure 7:**
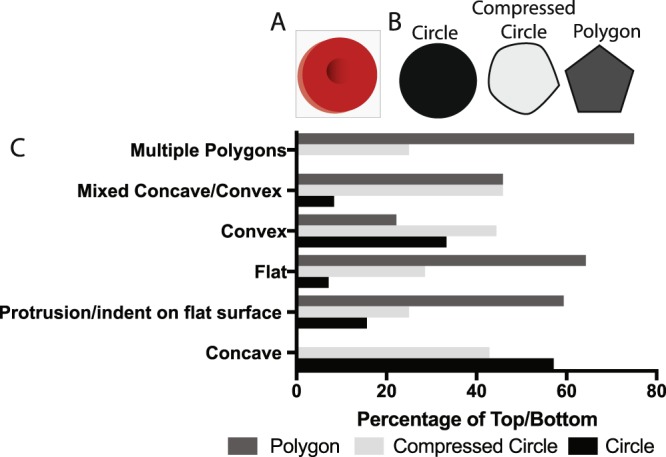
Analysis of structure of top and bottom surfaces of polyhedral-like erythrocytes. Three-dimensional reconstructions of intermediate forms of erythrocytes from contracted whole blood clots were assessed by structural characterization of (**A**) the cross-sections perpendicular to the height shown as a blue box on the red erythrocyte (**B**) with the appearance of a circle, compressed circle, or polygon. (**C**) The top and bottom surfaces were then quantified based on the appearance of cross-sections perpendicular to the height.

The intermediate forms of erythrocytes are exposed to compressive forces like the polyhedrocytes, but their shapes are not as clearly defined. As one method to assess the successive shape changes of the erythrocytes, we looked at the two-dimensional cross-section of the cells perpendicular to their shortest axis, determined through the analysis of three-dimensional reconstructions of individual cells from confocal images. These cross-sections represented various closed shapes (Fig. 7A). For example, the cross-section of a biconcave cell would be a circle. In comparison, the cross-section of a polyhedrocytes was a polygon (Fig. 7B). The cross-section of the intermediate forms could be characterized as a circle, a polygon, or something in between the two, which we refer to as a “compressed circle”.

To visualize the intermediate forms of erythrocytes, we have characterized the “top” and “bottom” of the individual erythrocyte relative to the two-dimensional cross-sections (Fig. 7A,B). The top and bottom of the cells were characterized as concave, convex, mixed concave/convex, flat, protrusion/indent on flat surface or multiple polygons. These characteristics are detailed in Table 2 and Supplemental Fig. 6. The majority of the cells with a concave top or bottom were associated with a circular cross-section, and none were associated with a polygonal cross-section. Conversely, the multiple polygons were more frequently associated with a polygonal cross-section and not associated with a circular cross-section (Fig. 7C). Cells with a compressed circle cross-section had a variety of every category of shape (Fig. 7C).

**Table 2:** Morphological characteristics of top and bottom of intermediate erythrocytes observed with confocal microscopy.

Classification	Inclusion Criteria
Concave	A clear dimple in the middle of the cell
Flat	The surface was flat
Convex	The surface curved outward and was rounded (hemisphere)
Mixed Concave/Convex	Part of the surface was convex, going above the cross-section and part was concave, going below the cross-section
Protrusion/indentation on flat surface	The surface was otherwise flat except a small area where there was either a protrusion or indentation
Multiple polygons	The surface was flat and made up of at least two intersecting polygons

### Quantitative geometrical characterization of polyhedrocytes

**Figure 8:**
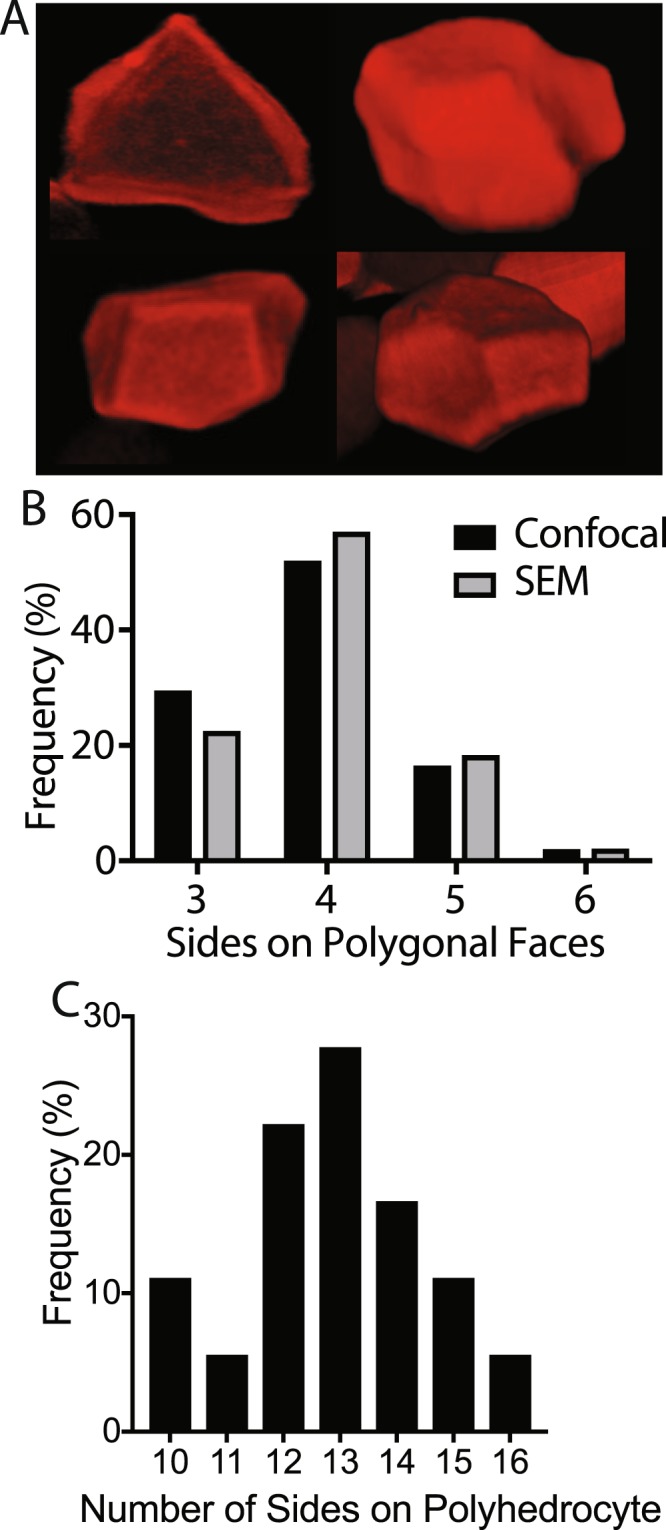
Quantification of the 3D images of polyhedral erythrocytes. Confocal images of polyhedral erythrocytes in contracted blood clots (**A**) were quantified for the content of 3-, 4-, 5- and 6-sided polygonal faces (**B**). N > 140 polygons analyzed. (**C**) The total number of sides on individual polyhedrocytes in contracted blood clots were counted for 3D images on 18 individual cells.

Since scanning electron microscopy images allow visualization of the faces of the polyhedrocytes, we assessed the distribution of polygonal faces and determined the types and frequency. All the polygons were either triangles, quadrilaterals, pentagons, or hexagons, with the over 50% of the polygons being quadrilaterals (Fig. 8). Confocal microscopy was used to visualize the three-dimensional structure of the polyhedrocytes (Supplement Fig. 7) and likewise the polygons that make up the polyhedrocytes were quantified. The distribution of polygons corresponded to what was observed with scanning electron microscopy (Fig. 8B), confirming that, in spite of the artifacts and limitations of both types of microscopy, both can be used to visualize the structures of the erythrocytes. Importantly, where scanning electron microscopy is limited to two-dimensional views of these cells, confocal microscopy allows for an examination of the whole three-dimensional structure of the polyhedrocytes. With computational three-dimensional reconstruction, we found that the frequency of various polygonal sides of the polyhedrocytes was normally distributed, consisting of 10–16 sides with a median of 13 faces (Fig. 8C, Table 3). The number of sides on the polyhedrocytes were normally distributed around the median as determined by a normality test. Interestingly, there was no correlation between the total number of sides that made up the polyhedrocyte and the distribution of faces, but there was a correlation between the relative percentages of the polygonal faces (Table 4). It was determined that if there were more faces with 3 sides, then there were fewer faces with either 4 or 5 sides.

**Table 3:** Number of polygonal faces based on total number of sides from 3D confocal microscopy of individual polyhedrocytes in contracted blood clots.

Total sides	Polygonal faces			
	Triangle	Quadrilateral	Pentagon	Hexagon
10	4	5	1	0
10	3	6	1	0
11	0	7	4	0
12	2	9	1	0
12	5	7	0	0
12	3	6	3	0
12	4	7	1	0
13	2	8	3	0
13	1	8	4	0
13	1	9	3	0
13	3	9	1	0
13	2	8	3	0
14	6	4	4	0
14	10	4	0	0
14	4	8	2	0
15	7	7	1	0
15	2	10	1	2
16	5	10	1	0

**Table 4:** Correlation coefficients between total sides in polyhedron and polygonal faces in individual polyhedrocytes formed during blood clot contraction.

	Total sides	Triangle	Quadrilateral	Pentagon	Hexagon
Total sides		0.246	−0.208	−0.137	0.036
Triangle	0.247		−0.775**	−0.642**	−0.173
Quadrilateral	−0.208	−0.775**		0.0125	0.362
Pentagon	−0.137	−0.642**	0.012		−0.166
Hexagon	0.036	−0.173	0.362	−0.166	

**p < 0.01.

## Discussion

We have described and analyzed a new type of erythrocytes, polyhedral-like erythrocytes, using a variety of imaging techniques. This shape of erythrocytes is a unique morphological form, different from those that have been described for centuries, since the first microscopic description by van Leeuwenhoek. It is important to note that this is a naturally occurring morphological form of erythrocytes that happens not only *in vitro* but has also been observed *in vivo*, thus comprising an objective structural signature of intravital contraction of clots and thrombi^10,23–25^.

We can relate our findings on polyhedrocytes to the polyhedra that have been observed in nature since at least the time of Plato in a variety of cells, tissues, viruses, foams and non-biological materials. Polyhedra have been described in live tissues, viruses, foams and metals; the tessellated networks that polyhedra form have been shown to occur as a means to minimize energy or to minimize surface area in filling space. There are similarities between grains in solidifying metals, which are largely determined by surface tension, and the packing of soap foams, where the structure is determined by surface tension and space-filling considerations^32^. These polyhedra have on average 12.5 faces with each face having on average 5 edges^32^. Interestingly, these values are similar to those that we have observed for the polyhedrocytes. Moreover, the visual representations of grain shapes of aluminum bear a striking resemblance to the cells observed here. Importantly, through the use of three-dimensional reconstruction and quantification of individual polyhedrocytes, we determined that polyhedrocytes contain an average of 13 faces, with each face having between 3 and 6 sides.

The biconcave shape and deformability of the erythrocyte is important for the cell to be able to fit through capillaries smaller than its size^33^. It is striking that the polyhedrocytes had a sphericity not different from the biconcave cells, with a value of ~0.8. The oblate and prolate profile of the polyhedrocytes reveals that it takes on an ellipsoidal form. Importantly, it has been shown that red cells in other species with an ellipsoidal form, such as from llamas and camels, are still able to bend and have a higher surface area than expected while still being able to maintain a low volume^34–36^. It is also important that neither the surface area nor the volume changes during the transition between biconcave erythrocytes and polyhedrocytes, since a change in surface area would mean a change in amount of membrane, while a change in volume would require the cellular cytoplasm to increase or decrease^37^.

In general, a tessellated polyhedral network has been shown to arise due to a variety of forces, resulting in shape change from spheres to polyhedra with a wide range of number of faces^38–41^. The erythrocyte’s cytoskeleton allows the cell to undergo large deformations over a wide range of forces^42^, and have been shown to have an elastic modulus of 3.1 × 10^7^ dyn/cm^2^ prior to lysis^43^. Here, we determined that shape change of the erythrocytes is linked to the application of mechanical forces/pressure of the order of 100 dyn/cm^2^ (Figs 2, 3), which is much smaller than the range of natural stresses that clots and thrombi experience as a result of platelet contractility^31^. This finding is biologically meaningful because it corresponds to the influence of platelet count and thrombin activity on the extent of clot contraction^5^, where the platelet-generated contractile forces compress the deformed erythrocytes into the core of the clot. To emphasize this dependence on pressure, we propose the use of the term “piezocytes” (derived from the Greek *piezein*, which means to squeeze or press) to characterize erythrocytes that have undergone a shape change resulting from the application of platelet-generated contractile/compressive force. “Piezocytes” would designate all mechanically deformed erythrocytes including polyhedrocytes as well as multiple intermediate forms without the strict geometry of a polyhedron.

Polyhedrocytes as a morphological marker of clot contraction *in vivo*^11,23,25^ may be useful in forensic medicine or the study of pathological conditions as a patho-morphological sign of a relatively mature thrombus or thrombotic embolus that underwent intravital contraction. We show here that polyhedrocytes or intermediate/polyhedral cells make up ∼90% of erythrocytes present in thrombi extracted from deep vein thrombosis patients (Fig. 2). More importantly, the structure and biological properties of compressed erythrocytes can potentially influence the course and outcomes of thrombosis through a number of pathogenic mechanisms. *First*, it has been suggested that clot contraction can influence the restoration of blood flow past otherwise obstructive thrombi^44^. Although the severity of thrombosis is largely determined by the diameter and location of the occluded vessels, the obstructiveness of the uncontracted/contracted thrombi has the potential to influence the impairment of blood flow. Poiseuille’s Law means that a reduction in the cross-sectional area of the vessel can greatly decrease the blood flow through the vessel and increase the shear forces on the thrombi. *Second*, clot contraction has been shown to be reduced in patients with pulmonary emboli compared to those with isolated venous thrombi^12^, which points to the fact that compacted erythrocytes may have the potential to reduce the embologencity of the thrombi, while less compacted cellular masses may be more embologenic. *Third*, the compactness of the thrombi ultimately has the potential to influence the dissolution of the clot, likely due to the influence of compacted erythrocytes on reducing the permeability of the clot for thrombolytic enzymes^10^. *Fourth*, the deformability of erythrocytes has effects in diseases that are predisposed to thrombotic events, such as sickle cell disease, malaria, diabetes, and high cholesterol, suggesting that altered erythrocyte deformability could contribute to bleeding or thrombotic responses in these patient populations. In all of these conditions, erythrocytes have been found to be markedly stiffer than in healthy subjects. In sickle cell disease patients and related conditions, the increased stiffness of erythrocytes arises from the intracellular polymerization of mutated hemoglobin S^45–47^, whereas in metabolic conditions, increases in erythrocyte stiffness can arise due to oxidative stress and/or increased membrane cholesterol^30,48^. In addition, the degree of erythrocyte compression may help or interfere with the mechanical removal of thrombi during interventional or surgical thrombectomy. Tightly compressed erythrocytes may also improve hemostasis in the wound and provide a natural barrier for pathogens and toxins^23^.

In conclusion, in this study we characterized the shape change of erythrocytes following the application of mechanical forces that occur during the blood clot contraction process. Formation of polyhedral erythrocytes as a result of platelet-driven mechanical deformation is another piece of evidence for the involvement of erythrocytes in hemostasis and thrombosis. Determination of the biological sequelae of polyhedrocyte formation is a new avenue of research in the field of hemostasis and thrombosis. Polyhedral-like erythrocytes have the potential to affect strongly the physical and biological properties of intravascular blood clots and thrombi with various degrees of compaction.

## Materials and Methods

### Sample preparation

Venous blood from healthy subjects was collected in 3.2% sodium citrate (9:1 vol:vol) following informed consent in accordance with the guidelines set forth by an Institutional Review Board at the University of Pennsylvania. Collection of blood and experimental protocols were in compliance with those approved by the Institutional Review Board at the University of Pennsylvania. Citrated whole blood was centrifuged at 175 g to separate erythrocytes and platelet-rich plasma (PRP). PRP was further centrifuged at 3,000 g to obtain platelet-poor plasma (PPP) and erythrocytes were washed in phosphate-buffered saline three times. Whole blood or reconstituted samples (made by mixing PPP, PRP, and washed erythrocytes) were clotted with simultaneous platelet activation by adding 1 U/ml human thrombin (Sigma-Aldrich) and 2 mM CaCl_2_ (final concentrations) unless otherwise noted. Clots formed within about 1 minute were allowed to contract for 30–60 minutes. The formation and contraction of clots was carried out at 37 °C to prevent potential effects of temperature modulation on thrombin activity, platelet contractility, and RBC membrane rigidity.

### Thrombi preparation

This study was approved by the Ethical Committee of the Interregional Clinical Diagnostic Center (Kazan, Russian Federation) and informed written consent was obtained from patients admitted to the Department of Vascular Surgery. Thrombi were extracted from 9 patients with thrombosis of the femoral vein during surgical thrombectomy, they were rinsed with saline and immediately fixed in 2% glutaraldehyde to prepare them for scanning electron microscopy.

### Histological examination

Citrated whole blood was activated with 1 U/ml thrombin and 2 mM CaCl_2_ (final concentrations) and allowed to clot and further contract for 60 minutes at room temperature. To prevent clot contraction, in some samples the 10-fold volume of fixative was added to the clotted blood within 2 minutes after addition of thrombin. The fresh uncontracted or contracted clots prepared in parallel from the same blood sample were fixed in 10% neutral buffered formalin, washed in water, cut, dehydrated serially in ascending concentrations of ethanol, and embedded in paraffin. 4-µm-thick sections were stained with hematoxylin and eosin and with picro-Mallory’s stain using standard protocols.

### Transmission electron microscopy

Fresh uncontracted or contracted clots prepared as for histological examination were fixed in 2% glutaraldehyde (final concentration) in 50 mM cacodylate buffer, pH 7.4, containing 150 mM NaCl and then added to a 2% aqueous OsO_4_ solution at a 1:1 volume ratio. Samples were dehydrated in ascending concentrations of ethanol and then added to propylene oxide. The samples were stored in a 1:1 propylene oxide/Epon resin overnight, then sectioned using an ultramicrotome LKB-III, stained with uranyl acetate for 2 hours at room temperature, and then exposed to lead citrate for 10 minutes. Sections were imaged using a JEM-1200 EX transmission electron microscope (Jeol, Japan).

### Scanning electron microscopy

Reconstituted or whole blood contracted clots (prepared as for histological examination) as well as a sediment of erythrocytes (settled or centrifuged), and extracted venous thrombi were fixed in 2% glutaraldehyde (final concentration) in cacodylate buffer, pH 7.4, containing 150 mM NaCl. Samples were dehydrated in ascending concentrations of ethanol, then dried overnight in hexamethyldisilazane. The samples were sputter-coated with a 15-nm thin layer of gold-palladium. Micrographs were obtained using a FEI Quanta 250FEG scanning electron microscope (FEI, Hillsdale, Oregon). The cells were analyzed such that three-five representative images were selected for each experimental condition. In each image the erythrocytes were characterized as biconcave (smooth, round, clear dimple), intermediate biconcave (smooth, round, slight or no dimple), intermediate polyhedral (visible edges, no dimple), and polyhedral (defined edges, flat faces). The full description of characterization criteria can be found in Table 1 and Supplemental Fig. 2. Erythrocytes were excluded from analysis if they were not completely in the image frame, out of focus, or blocked by other erythrocytes. Each erythrocyte was characterized manually in a blinded manner. Total number of erythrocytes, number of erythrocytes in each category, and percentages were calculated for individual images. The number of erythrocytes analyzed depended on the number of cells per image and the number of images analyzed, and at least 100 erythrocytes were analyzed per sample. To prevent skewing based on total number of cells analyzed per experimental condition that data is represented as the average percentage of each category per individual image. Comparisons were then made between experimental groups.

### Confocal microscopy

For confocal microscopy, isolated erythrocytes were labeled with DiD (Thermo Fischer, Waltham, Massachusetts) according to the manufacturer’s directions; then 10 µl of labeled sedimented (packed) erythrocytes was mixed with 90 µl of unlabeled sedimented erythrocytes and 100 µl of freshly obtained platelet-rich plasma. Clotting was initiated with 1 U/ml thrombin and 2 mM CaCl_2_ and the activated reconstituted sample was quickly transferred to an 8-well u-Slide (Ibidi, Martinsreid, Germany). Clots formed within about 1 minute were allowed to contract for 30 minutes at 37 °C and then fixed with 2% glutaraldehyde (final concentration) in cacodylate buffer, pH 7.4, containing 150 mM NaCl. Images of contracted clots were collected using a Zeiss LSM 710 or Leica SP8 confocal microscope through excitation at 633 nm (LSM 710) or 638 nm (SP8). Apochromat 63 × /1.4 oil DIC objective was used with a 6–10 μs pixel dwell time and a voxel size of 70 × 70 × 170 nm.

Three-dimensional reconstruction of confocal microscopy stacks of images was carried out using the Volocity (PerkinElmer, Waltham, Massachusetts) and Imaris (Bitplane, Zurich, Switzerland) software. Erythrocytes were reconstructed using contour surface generation based on the fluorescence intensity of the cells. All image analysis was conducted using the Imaris software. Erythrocytes were detected using the local contrast mode which applies a Gaussian filter to determine the intensity of each voxel. This allowed for information about cell size, volume, and shape to be obtained.

### Statistical Analysis

All analyses were completed using GraphPad Prism 6.0. A one-way ANOVA or a Student’s *t*-test with an alpha value of 0.05 were used to compare differences between samples as described for each result. All data is represented as a mean ± SEM unless otherwise noted. A D’Agostino & Pearson normality test was used to determine the distribution of faces in polyhedral erythrocytes. Spearmen correlation analysis was used to assess connections between the number of polygonal faces on the polyhedral erythrocytes.

## Electronic supplementary material

Supplementary Figures
