# Supplementary material for: Shape changes of erythrocytes during blood clot contraction and the structure of polyhedrocytes
Source: Sci Rep. 2018 Dec 17;8:17907. doi: 10.1038/s41598-018-35849-8 (PMC6297136; doi:10.1038/s41598-018-35849-8)
Supplement: Supplementary file 1 — Supplementary Figures [file 41598_2018_35849_MOESM1_ESM.docx]

**Shape changes of erythrocytes during blood clot contraction and the structure of polyhedrocytes**

*Short title*: Polyhedral erythrocytes in contracted blood clots

Valerie Tutwiler^1^, Alexander R. Mukhitov^1^, Alina D. Peshkova^2^, Giang Le Minh^2^, R. R. Khismatullin^3^, Jacqueline Vicksman^1^, Chandrasekaran Nagaswami^1^, Rustem I. Litvinov^1^, and John W. Weisel^1^

^1^Department of Cell and Developmental Biology, University of Pennsylvania School of Medicine, Philadelphia, PA USA ^2^Institute of Fundamental Medicine and Biology, Kazan Federal University, Kazan, Russian Federation ^3^Department of General Pathology, Kazan State Medical University, Kazan, Russian Federation

*Correspondence:*

John W. Weisel, PhD

Department of Cell & Developmental Biology

School of Medicine

University of Pennsylvania

421 Curie Blvd

BRB II/III, Room 1154

Philadelphia PA 19104-6058

USA

Tel: 215-898-3573

Fax: 215-746-8781

weisel@pennmedicine.upenn.edu

**Supplemental Figures**


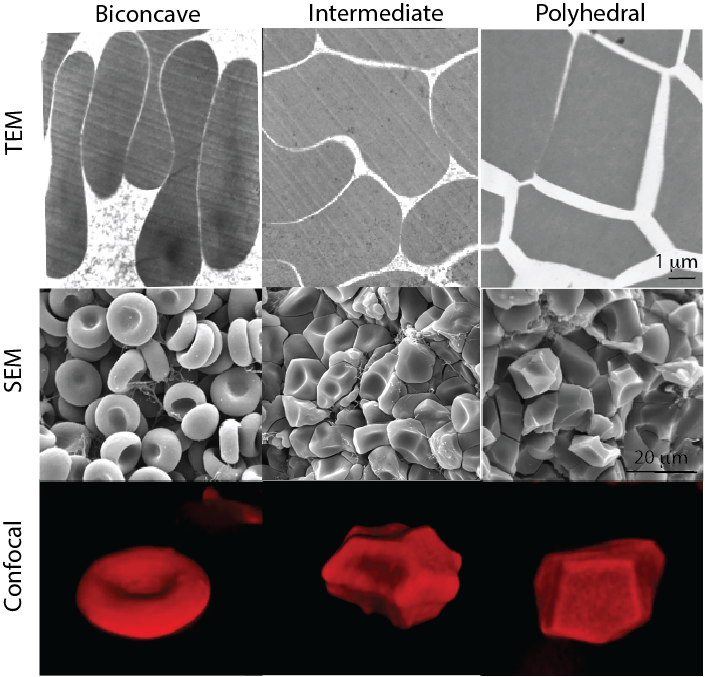


**Supplemental Figure 1. Visualization of biconcave, intermediate forms, and pure polyhedral erythrocytes using** transmission electron microscopy (TEM), scanning electron microscopy (SEM), and confocal microscopy. Scale bar is 1 µm for TEM, 25 µm for SEM, and 10 µm for confocal.


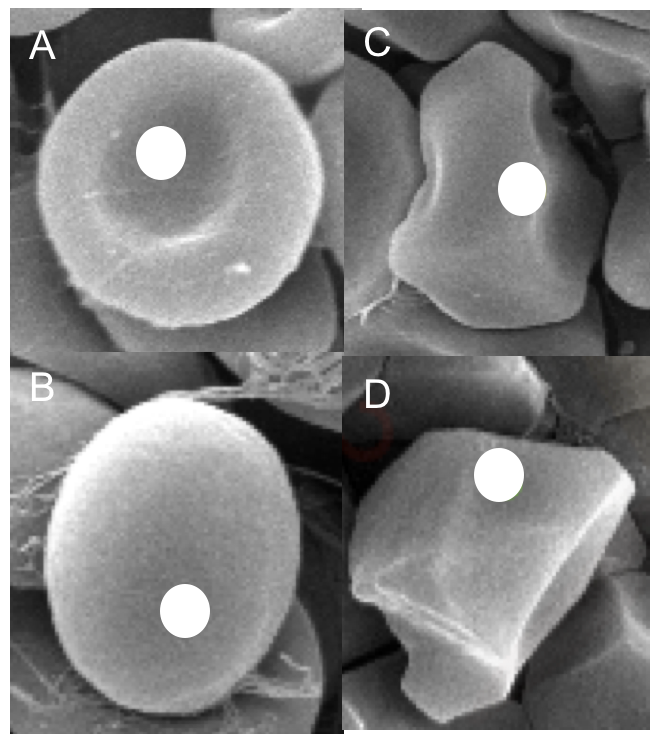


**Supplemental Figure 2. Representative images of erythrocyte characterization criteria.** Scanning electron microscopy images were characterized for (A) biconcave, (B) intermediate biconcave, (C) intermediate polyhedral, and (D) polyhedral. Example erythrocytes are marked with a white dot.


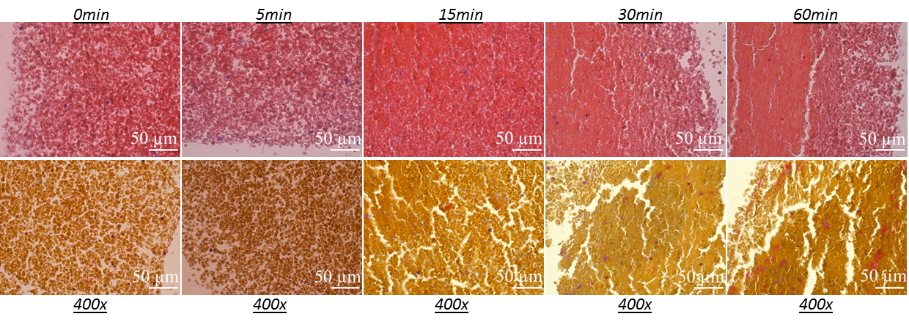


**Supplemental Figure 3. Time-dependent histological images of contracting blood clots showing erythrocyte packing and shape change.** Top row shows hematoxylin and eosin staining and the bottom row shows picro-Mallory staining. Scale bar is 50 μm.


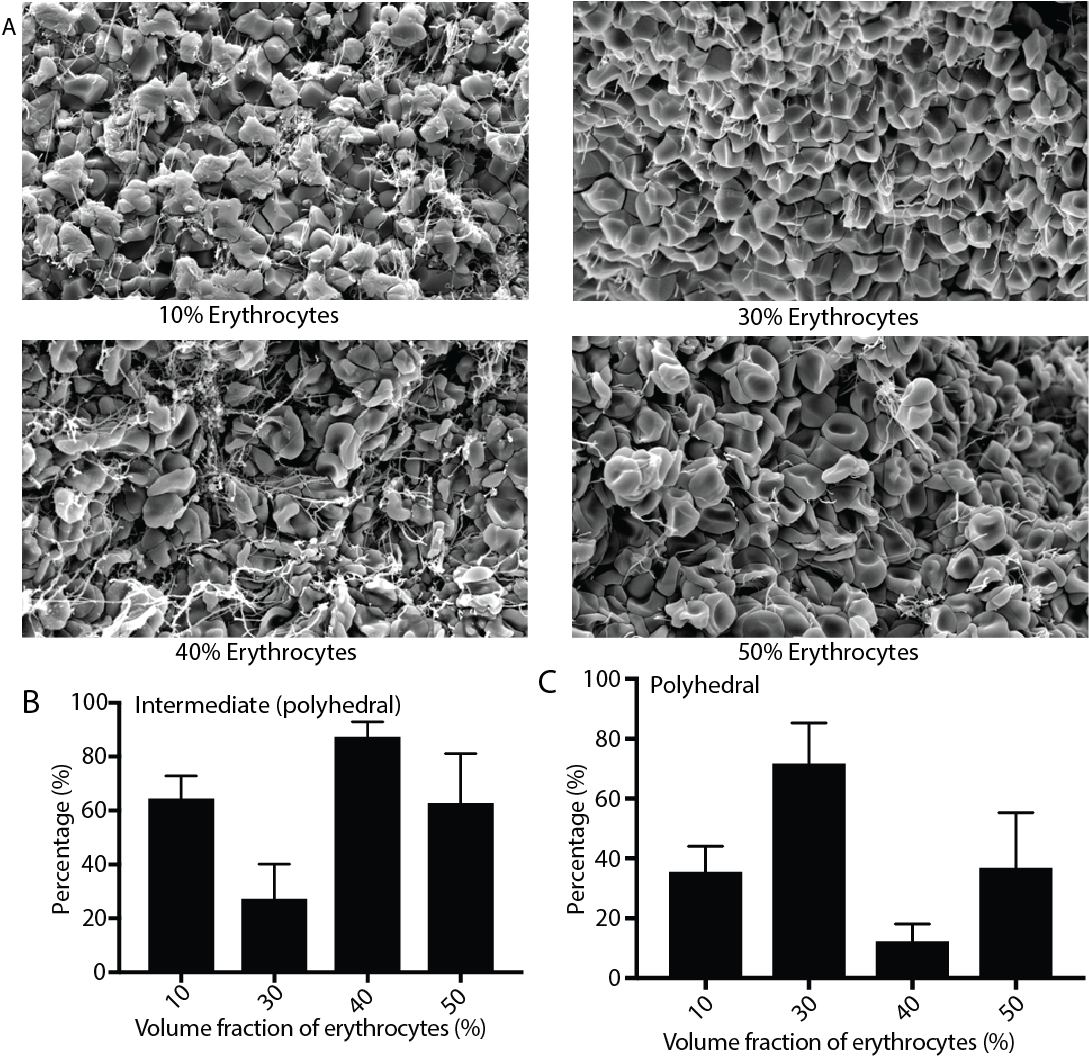


**Supplemental Figure 4. Influence of volume fraction of erythrocytes on formation of polyhedral-like erythrocytes in contracting blood clots.** Reconstituted whole blood samples with varying volume fractions of erythrocytes from 10% to 50% were activated with 1 U/ml thrombin and 2 mM CaCl_2_ and allowed to contract and then imaged using scanning electron microscopy. (**A**) Representative scanning electron microscopy images showing that the extent of polyhedral-like deformation of erythrocytes is independent of their volume fraction. Scanning electron microscopy images were quantified for the percentage of (**B**) intermediate (polyhedral), and (**C**) pure polyhedral cells. Statistical analysis was completed using a one-way ANOVA with an alpha value of 0.5. Between 300 and 1000 erythrocytes were analyzed for each volume fraction of erythrocytes. Scale bar is 10 µm.

**Width (um)**

**Supplemental Figure 5. Width and height measurements for erythrocytes of various shapes determined using confocal microscopy.**

**
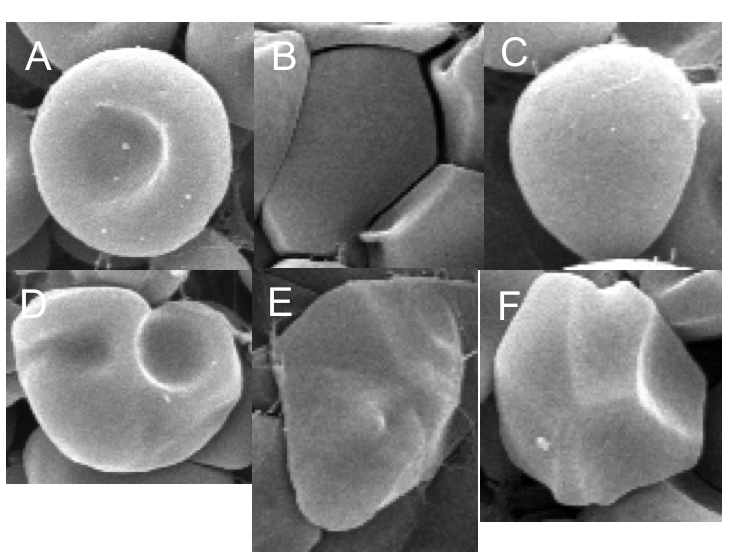
**

**Supplemental Figure 6. Representative images for morphological characteristics of top and bottom of intermediate erythrocytes.** Morphological characteristics were observed using scanning electron and confocal microscopy. The top and bottom of the intermediate erythrocytes were characterized as (A) concave, (B) flat, (C) convex, (D) mixed concave/convex, (E) flat with a protrusion/indent, and (F) multiple polygons.

**
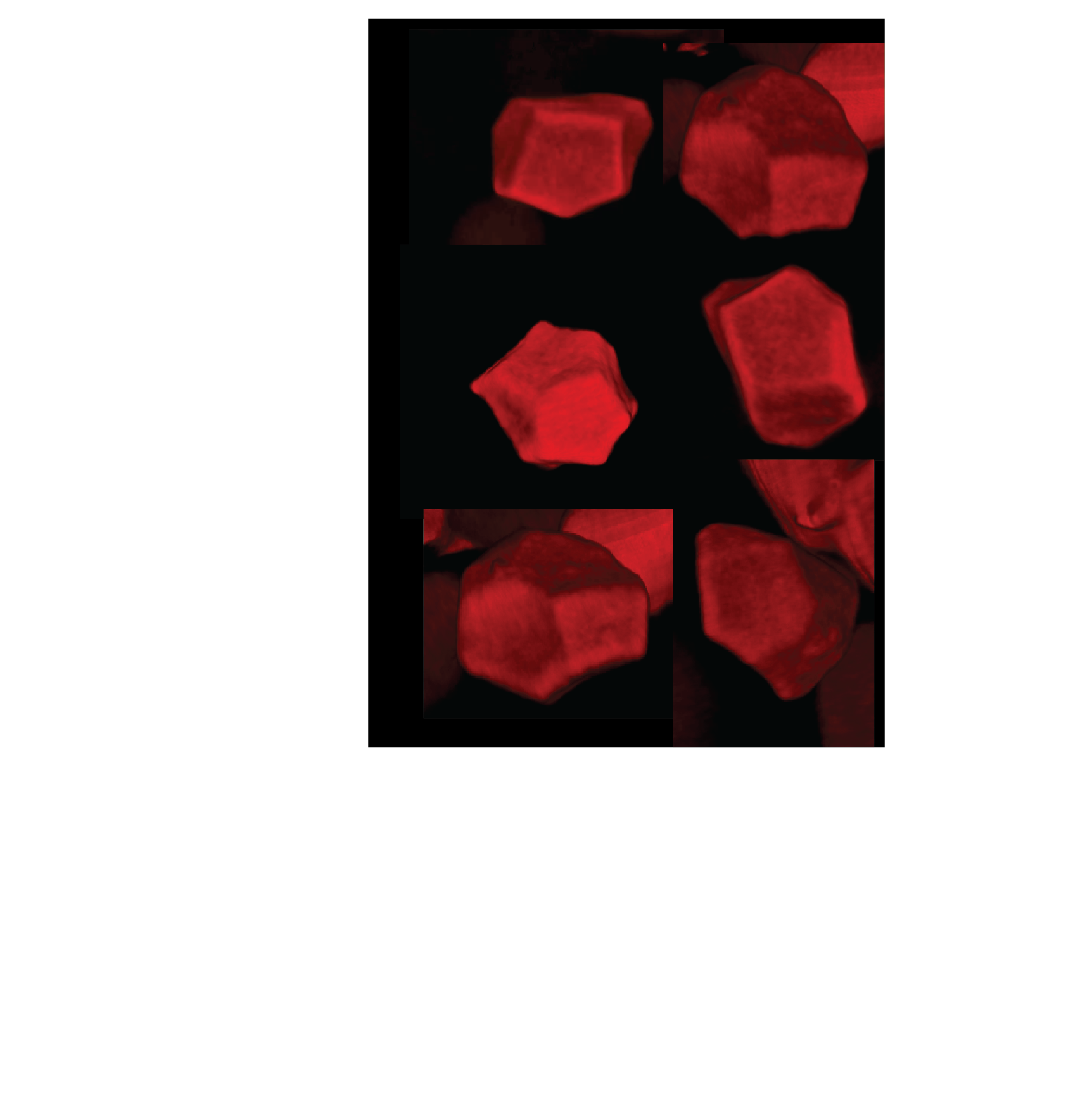
**

**Supplemental Figure 7. Examples of 3D confocal microscopy images of polyhedral erythrocytes**
